# EuReCa ONE – 27 Nations, ONE Europe, ONE Registry: a prospective observational analysis over one month in 27 resuscitation registries in Europe – the EuReCa ONE study protocol

**DOI:** 10.1186/s13049-015-0093-3

**Published:** 2015-01-24

**Authors:** Jan Wnent, Siobhán Masterson, Jan-Thorsten Gräsner, Bernd W Böttiger, Johan Herlitz, Ruud W Koster, Fernando Rosell Ortiz, Ingvild Tjelmeland, Holger Maurer, Leo Bossaert

**Affiliations:** Department of Anaesthesiology and Intensive Care Medicine, University Medical Centre Schleswig-Holstein, Campus Luebeck, Ratzeburger Allee 160, 23838 Luebeck, Germany; Discipline of General Practice, National University of Ireland Galway and Department of Public Health Medicine HSE, St. Conal’s Hospital, Letterkenny, Co. Donegal Ireland; Department of Anaesthesiology and Intensive Care Medicine, University Medical Centre Schleswig-Holstein, Campus Kiel, Kiel, Germany; Department of Anaesthesiology and Intensive Care Medicine, University Hospital of Cologne, Cologne, Germany; University of Borås, Sahlgrenska University Hospital, Göteborg, Sweden; Academisch Medisch Centrum, Department of Cardiology, Amsterdam, Netherlands; Empresa Pública de Emergencias Sanitarias, Almería, Spain; Nasjonal kompetansetjeneste for prehospital akuttmedisin (NAKOS), Oslo, Norway; University of Antwerp, Belgium and European Resuscitation Council, Niel, Belgium

**Keywords:** Out-of-hospital-cardiac arrest, Resuscitation registry, EuReCa ONE, Cardiopulmonary resuscitation, European resuscitation council, European registry of cardiac arrest (EuReCa), Resuscitation outcome

## Abstract

**Background:**

There is substantial variation in the incidence, likelihood of attempted resuscitation and outcomes from out-of-hospital cardiac arrest (OHCA) across Europe. A European, multi-centre study provides the opportunity to uncover differences throughout Europe and may help find explanations for these differences. Results may also have potential to support the development of quality benchmarking between European Emergency Medical Services (EMS).

**Methods/Design:**

This prospective European study involves 27 different countries. It provides a common Utstein-based dataset, data collection tool and a common data collection period for all participants.

Study research questions will address the following: OHCA incidence in different European regions; incidence of cardiopulmonary resuscitation (CPR); initial presenting rhythm in patients where bystanders or EMS start CPR or any other resuscitation intervention; proportion of patients with any return of spontaneous circulation (ROSC); patient status at the end of pre-hospital treatment i.e. ROSC at handover to hospital, ongoing CPR, dead; proportion of patients still alive 30 days after OHCA; proportion of patients discharged alive from hospital.

All patients who suffered an OHCA during October 2014 and were attended and/or treated by an EMS and documented in one of the participating registries will be included in the study. Each National Coordinator is responsible for data collection and quality control in his/her country and will transfer unprocessed anonymised data via secure electronic transfer.

Descriptive analysis will be performed at European, national and registry level. For endpoints like ROSC, admission or survival, multivariate logistic regression analysis will be performed.

**Discussion:**

Documenting differences in epidemiology, treatment and outcome in out-of-hospital cardiac arrest throughout Europe is a first step in finding explanations for these differences. Study results might also support the development of quality benchmarking between Emergency Medical Services (EMS) which in turn will facilitate initiatives to improve OHCA outcome in Europe.

**Trial registration:**

The EuReCa ONE Study is registered by ClinicalTrials.gov National CoordinatorT02236819).

## Background

Cardiovascular disease is the leading cause of death in Europe, accounting for 4.1million deaths per year and approximately 37% of all deaths in patients younger than 75 years of age [1]. There is considerable variation in the incidence of out-of-hospital cardiac arrest (OHCA) between European countries and communities and the incidence of resuscitation attempts also varies widely (38-86/100,000 inhabitants/year) [2,3]. Reasons for variation in incidence may include differences in cardiovascular disease prevalence, lifestyle and nutritional behaviour, differences in the structure and deployment of emergency medical services (EMS) and variation in treatment options in receiving hospitals [4]. It has been suggested that in recent years, developments in pre-hospital as well as early in-hospital treatment might have increased the ratio of admission to hospital and survival [5]. Differences in data definitions, methods of data collection and the quality of data reported may also play a role in variability [6].

Numerous research projects have been undertaken to improve the outcome after out-of-hospital cardiac arrest. Nevertheless it is assumed that there is potential for further improvement. To uncover factors associated with better outcome, more knowledge about the incidence, management and outcomes from out-of-hospital cardiac arrest is required [7].

Reliable and robust data must be available to support changes in the current approach to cardiac arrest and to improve quality of care. This prospective European study provides a common dataset, common data collection tool and a single data collection period for all participants [3,8].

This study aims to provide a one-month snapshot of the epidemiology, treatment and the short term outcomes for patients who suffer an out-of-hospital cardiac arrest in Europe. The prospective collection of this data took place during October 2014 and the process of submitting data for processing and analysis commenced in January 2015.

The EuReCa ONE study is funded by the European Resuscitation Council (ERC) and the national resuscitation registries. As the main funder, the European Resuscitation Council is the legal owner of the European Registry of Cardiac Arrest (EuReCa). The study is governed by a Steering Committee from the European Resuscitation Council and is administered and conducted by a European Study Management Team.

## Methods/Design

Every patient who suffered a cardiac arrest during October 2014, that occured in any location other than a hospital capable of providing emergency resuscitation, and was attended and/or treated by an Emergency Medical Service and documented in one of the participating registries will be included in the study. Patients were included regardless of arrest aetiology, initial arrest rhythm, age or gender. These inclusion criteria include every patient who received chest compressions and/or defibrillation by the EMS or by bystanders before arrival of the EMS with continued resuscitation by the EMS; by a bystander before arrival of the EMS that was immediately stopped by EMS and every resuscitation attempt by bystander with ROSC before the arrival of the EMS. Patients found or declared dead by the EMS for any reasons are also included.

Every National Coordinator from each participating country was responsible for obtaining ethical approval unless a documented waiver was acceptable in that country. Participants are prohibited from submitting data unless a documented waiver or ethical approval is submitted to the Study Management Team. As only anonymised data will be reported and the data is recorded as part of routine data, a requirement for patient consent was not expected. It is however the responsibility of each National Coordinator to ensure that patient consent is not required in his/her jurisdiction.

Since 1991, the Utstein dataset has provided a widely accepted and uniform template for the collection of out-of-hospital cardiac arrest data and was updated in 2004 [9]. The study dataset was developed in accordance with Utstein criteria and it was attempted to ‘future-proof’ the dataset in accordance with the 2014 Utstein revision (not published at time of data collection) [10]. Every participating registry is required to ensure that they can comply with the nomenclature and data definitions described in the EuReCa ONE dataset (see appendix). Variables are divided into core and supplementary data. In order to participate in EuReCa ONE, all registries must supply data that fulfils at least the core dataset otherwise the registry cannot be included in the analysis. All data must be submitted to the EuReCa ONE database by one National Coordinator in each participating country. In order to ensure data security and patient privacy all data must be submitted in anonymised format.

**Figure 1:**
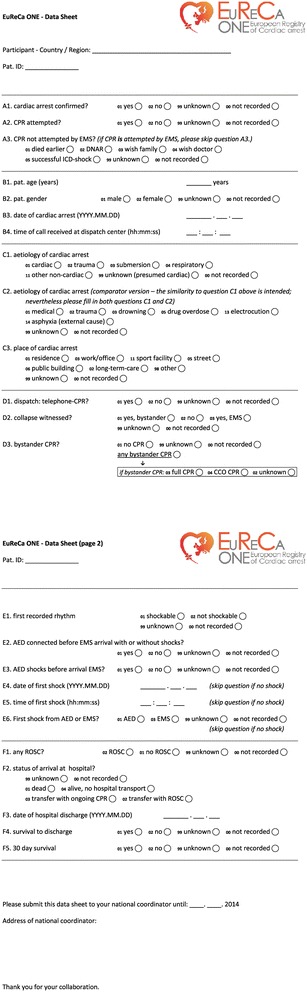
**EuReCa ONE - data sheet.**

The National Coordinator is responsible for the data collection in his/her country. Data will be extracted from existing national, regional or local resuscitation registries. Every case is to be transferred from the local registry onto an Excel-based database provided to each National Coordinator. In order to facilitate uniform national data collection, a datasheet (see Figure 1) was provided to all National Coordinators for dissemination to participating registries. National Coordinators also had access to a hotline telephone number during the data collection period so that questions and queries relating to data collection could be dealt with quickly and effectively.

National Coordinators must transfer unprocessed anonymised data. The transmission of aggregated data must only occur in cases where ethical approval for individual data transfer was not forthcoming.

The National Coordinator is responsible for quality control of the transferred data. Every participating registry must collect basic EMS data on the region including population served and size of the area covered. Data transmission to the EuReCa ONE database will use the https web protocol to ensure maximum electronic security during data transferring, processing and analysis. Data quality will be assessed by the Steering Committee and Study Management Team prior to analysis. Any anomalies or queries arising will be addressed to National Coordinators.

In order to achieve the aim of providing a one-month snapshot of the epidemiology, treatment and the short term outcomes of OHCA in Europe, the following research objectives were defined:

- What is the incidence of out-of-hospital cardiac arrest (OHCA) in different European regions?
- What is the incidence of any CPR (cardiopulmonary resuscitation) attempted in OHCA throughout Europe
- In those cases where resuscitation is attempted:
- What proportion of patients is in a shockable rhythm at the time of first rhythm analysis?
- What proportion of patients achieves return of spontaneous circulation (ROSC) at any stage during the pre-hospital resuscitation attempt?
- What is the status of patients at the end of the pre-hospital resuscitation attempt i.e. what proportion of patients are: handed over to a hospital Emergency Department or hospital department with returned spontaneous circulation; handed over to hospital care with ongoing CPR; pronounced dead prior to hospital handover
- What proportion of patients are still alive 30 days (whether in-hospital or discharged) after OHCA
- What proportion of patients are discharged alive from hospital?
- In adult patients who suffer a witnessed collapse (witnessed by bystanders), and with a shockable rhythm at time of first rhythm analysis with an event of suspected cardiac cause i.e. Utstein comparator group [11,12]:
- What is the proportion of patients who have spontaneous circulation at the time of handover to hospital care
- What is the proportion of patients still alive at 30 days (whether in-hospital or discharged) after their cardiac arrest event and what is the proportion of patients who are discharged alive from hospital?
- What factors predict the outcomes of ROSC at any stage during pre-hospital resuscitation, patient status at the end of the pre-hospital resuscitation attempt and survival at 30 days and discharge from hospital alive?

Statistical analysis of the data collected will be provided by a statistician with the German Resuscitation Registry (GRR)®. All variables collected will be uniformly checked prior to analysis. These checks will include range checks, cross checks, and plausibility checks. In cases where the integrity of data is questionable, queries will be sent to the National Coordinator in the respective country. Incidence rates will be calculated per 100,000 inhabitants per month. While it would be desirable to provide estimates of expected incidence per month for this study, a previous attempt to estimate incidence highlighted the variability that might be expected between European countries and jurisdictions, with estimates averaging 86.4/100,000 and ranging from 24-173.6/100,000 person years (3). This difficulty in producing reliable estimates of incidence means that the validity of this study will depend heavily on the quality of data submitted by each National Coordinator.

Descriptive analysis of patient demographics, case characteristics and treatment and outcome variables will be performed for the whole group as well as for each participating registry (or country) separately in order to investigate the degree of variability between countries and regions. For both categorical and continuous variables and 95% confidence intervals (CI 95) will be calculated. Non-overlapping CI 95 will be interpreted as significant.

For certain outcome variables i.e. ROSC at any stage pre-hospital, ROSC at handover to hospital care, survival to 30 days and survival to hospital discharge, multivariate logistic regression analysis will be performed in the whole dataset. Independent predictor variables selected from the Utstein Core Dataset where relevance has been proven by published data will be included in the regression model. The source of data (participating country, or registry) may be included in these analyses in order to adjust for local variations and the model will be evaluated for interactions, including country-specific effects. The core model will be applied separately for each participating registry, resulting in a range of effectiveness measures (odds ratio) for each predictor.

It is intended that the results of this study be presented at the European Resuscitation Conference (ERC) in October 2015, during which the 2015 Resuscitation Guidelines will be announced [13]. It is hoped that initial presentation of the study as part of this critical event will maximise the dissemination of EuReCa ONE results and promote the continuation of OHCA data collection and use across Europe. In order to reach the wider international audience, the study will also be submitted for publication. Following publication, individual countries and regions will be free to publish and disseminate local results to ensure maximum local impact.

## Discussion

This will be the first study to gather data from 27 different countries in which OHCA care and treatment is managed by multiple EMS, all with their own clinical practice guidelines. EMS organisational structures vary across regions and countries, with different type and levels of staffing, and variation in the number and type of response vehicles available. Training of emergency physicians and pre-hospital emergency practitioners (technicians or paramedics) varies across Europe. The EuReCa ONE study provides an ideal opportunity to develop an overview of the outcomes achieved in different EMS systems and to begin the process of quality benchmarking OHCA outcome across Europe.

It is acknowledged that collecting data for a single month only will limit the comparability of results with regions outside the study area. An important focus of this study however, is to engage EuReCa ONE participants in OHCA data collection using an agreed data set. It was felt that a short data collection timeframe would be attractive and manageable to the widest possible number of prospective participants and would allow a reasonably quick turnaround with results. The strategy of having a short, manageable data collection period has already succeeded in 27 European countries agreeing to take part in the study. It is hoped that if EuReCa ONE is successful, the foundation for more pan-European OHCA studies will be laid.

A European, multi-centre study provides the opportunity to uncover differences in epidemiology, treatment and outcome in out-of-hospital cardiac arrest throughout Europe and may help find explanations for these differences. This study is the starting point for a pan-European registry of OHCA that can highlight the differences in OHCA treatment and survival throughout Europe and provide a foundation for improving the outcome following OHCA in the European countries.
